# (2*E*)-3-(4-Bromo­phen­yl)-1-(3-chloro­phen­yl)prop-2-en-1-one

**DOI:** 10.1107/S1600536809053446

**Published:** 2009-12-16

**Authors:** Jerry P. Jasinski, Ray J. Butcher, B. Narayana, K. Veena, H. S. Yathirajan

**Affiliations:** a Department of Chemistry, Keene State College, 229 Main Street, Keene, NH 03435-2001, USA; b Department of Chemistry, Howard University, 525 College Street NW, Washington, DC 20059, USA; c Department of Studies in Chemistry, Mangalore University, Manalaganotri, 574 199, India; d Department of Studies in Chemistry, University of Mysore, Manasagangotri, Mysore 570 006, India

## Abstract

In the title compound, C_15_H_10_BrClO, the dihedral angle between mean planes of the bromo- and chloro-substituted benzene rings is 46.2 (2)° compared to 45.20 (9)° in the structure with the Cl substituent in the *meta* position of the aromatic ring. The dihedral angles between the mean plane of the prop-2-ene-1-one group and the mean planes of the 4-bromo­phenyl and 3-chloro­phenyl rings are 28.7 (5) and 24.2 (4)°, respectively. In the crystal, weak inter­molecular C—H⋯π inter­actions occur.

## Related literature

For a related structure, see: Ng *et al.* (2006 ▶).
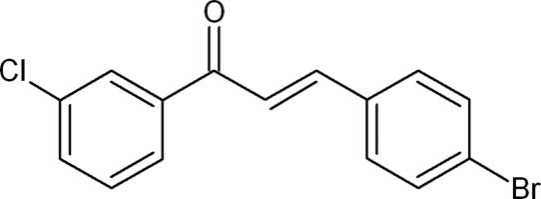


## Experimental

### 

#### Crystal data

C_15_H_10_BrClO
*M* *_r_* = 321.59
Triclinic, 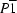

*a* = 5.9197 (8) Å
*b* = 7.3391 (11) Å
*c* = 14.8171 (17) Å
α = 101.929 (11)°
β = 94.371 (10)°
γ = 93.299 (11)°
*V* = 626.22 (15) Å^3^
*Z* = 2
Cu *K*α radiation
μ = 6.29 mm^−1^
*T* = 110 K
0.50 × 0.21 × 0.12 mm

#### Data collection

Oxford Diffraction Xcalibur diffractometer with a Ruby Gemini detector
Absorption correction: analytical (*CrysAlis RED*; Oxford Diffraction, 2007 ▶) *T*_min_ = 0.041, *T*_max_ = 0.344
3868 measured reflections
2432 independent reflections
2312 reflections with *I* > 2σ(*I*)
*R*_int_ = 0.037

#### Refinement

*R*[*F* ^2^ > 2σ(*F* ^2^)] = 0.058
*wR*(*F* ^2^) = 0.164
*S* = 1.07
2432 reflections
163 parameters
H-atom parameters constrained
Δρ_max_ = 1.78 e Å^−3^
Δρ_min_ = −1.29 e Å^−3^

### 

Data collection: *CrysAlis PRO* (Oxford Diffraction, 2007 ▶); cell refinement: *CrysAlis RED* (Oxford Diffraction, 2007 ▶); data reduction: *CrysAlis RED*; program(s) used to solve structure: *SHELXS97* (Sheldrick, 2008 ▶); program(s) used to refine structure: *SHELXL97*) (Sheldrick, 2008 ▶); molecular graphics: *SHELXTL* (Sheldrick, 2008 ▶); software used to prepare material for publication: *SHELXTL*.

## Supplementary Material

Crystal structure: contains datablocks global, I. DOI: 10.1107/S1600536809053446/bt5130sup1.cif

Structure factors: contains datablocks I. DOI: 10.1107/S1600536809053446/bt5130Isup2.hkl

Additional supplementary materials:  crystallographic information; 3D view; checkCIF report

## Figures and Tables

**Table 1:** Hydrogen-bond geometry (Å, °)

*D*—H⋯*A*	*D*—H	H⋯*A*	*D*⋯*A*	*D*—H⋯*A*
C2*A*—H2*AA*⋯*Cg*2^i^	0.95	2.97	3.588 (4)	124
C5*A*—H5*AA*⋯*Cg*2^ii^	0.95	2.84	3.463 (4)	124
C12*A*—H12*A*⋯*Cg*1^iii^	0.95	2.83	3.527 (4)	131

Symmetry codes: (i) 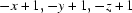; (ii) 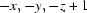; (iii) 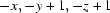. *Cg*1 is the centroid of the C1*A*–C6*A* ring and *Cg*2 is the centroid of the C10*A*–C15*A* ring.

**Fig. 1.**
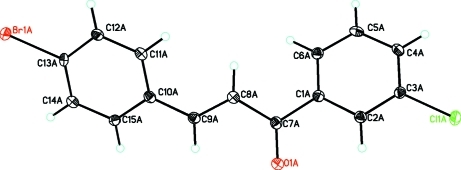
Molecular structure of the title compound, C15H10BrClO, showing the atom labeling scheme and 50% probability displacement ellipsoids.

**Fig. 2.**
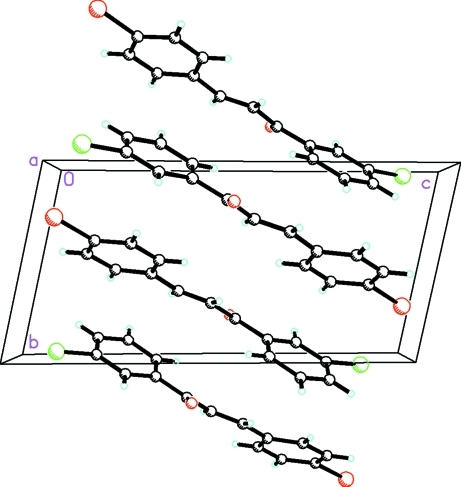
Packing diagram of the title compound, (I), viewed down the a axis.

**Fig. 3.**
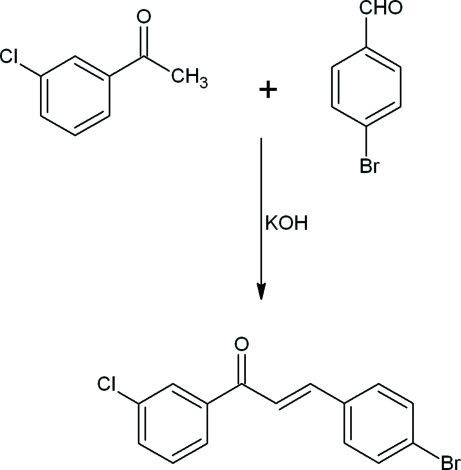
The formation of the title compound.

## supplementary crystallographic information

### Comment

In continuation of our interest in the synthesis and crystal structure determination of chalcones, the title chalcone, C_15_H_10_BrClO, is synthesized and its crystal structure is reported.

The title compound, (I), is a chalcone derivative with 4-bromophenyl and 3-chlorophenyl rings bonded at the opposite ends of a propenone group, the biologically active region (Fig.1). The dihedral angle between mean planes of the chloro and bromo substituted benzene rings is 46.2 (2)° compared to 45.20 (9)° (Ng *et al.* (2006)) and 46.70 (5)° for a similar related molecule. The angles between the mean plane of the prop-2-ene-1-one group and the mean planes of the 4-bromophenyl and 3-chlorophenyl rings are 28.7 (5)° and 24.2 (4)° and respectively. This compares to 20.66 (1)° and 24.54 (1)° in the similar structure. While no classical hydrogen bonds are present, weak intermolecular C–H···π-ring interactions are observed which contribute to the stability of crystal packing (Fig.2, Table 1).

### Experimental

50% KOH was added to a mixture of 3-chloroacetophenone (0.01 mol) and *p*-bromobenzaldehyde (0.01 mol) in 25 ml of ethanol (Scheme 2). The mixture was stirred for an hour at room temperature and the precipitate was collected by filtration and purified by recrystallization from ethanol. Single crystals were grown from ethyl acetate by slow evaporation method with the yield of the compound being 70% (m.p.412–414 K). Analytical data for C_15_H_10_BrClO: Found (Calculated): C %: 55.97 (56.02); H%: 3.09 (3.13).

### Refinement

All of the H atoms were placed in   calculated positions and then refined using the riding model with C—H = 0.95 Å, and with *U*_iso_(H) = 1.17–1.21*U*_eq_(C).

### Crystal data

**Table:**

C_15_H_10_BrClO	*Z* = 2
*M**_r_* = 321.59	*F*(000) = 320
Triclinic, *P*1	*D*_x_ = 1.706 Mg m^−^^3^
Hall symbol: -P 1	Cu *K*α radiation, λ = 1.54178 Å
*a* = 5.9197 (8) Å	Cell parameters from 3370 reflections
*b* = 7.3391 (11) Å	θ = 6.1–73.9°
*c* = 14.8171 (17) Å	µ = 6.29 mm^−^^1^
α = 101.929 (11)°	*T* = 110 K
β = 94.371 (10)°	Plate, colorless
γ = 93.299 (11)°	0.50 × 0.21 × 0.12 mm
*V* = 626.22 (15) Å^3^	

### Data collection

**Table:**

Oxford Diffraction Xcalibur diffractometer with a Ruby Gemini detector	2432 independent reflections
Radiation source: fine-focus sealed tube	2312 reflections with *I* > 2σ(*I*)
graphite	*R*_int_ = 0.037
Detector resolution: 10.5081 pixels mm^-1^	θ_max_ = 74.0°, θ_min_ = 6.1°
ω scans	*h* = −7→6
Absorption correction: analytical (*CrysAlis RED*; Oxford Diffraction, 2007)	*k* = −9→8
*T*_min_ = 0.041, *T*_max_ = 0.344	*l* = −18→18
3868 measured reflections	

### Refinement

**Table:**

Refinement on *F*^2^	Primary atom site location: structure-invariant direct methods
Least-squares matrix: full	Secondary atom site location: difference Fourier map
*R*[*F*^2^ > 2σ(*F*^2^)] = 0.058	Hydrogen site location: inferred from neighbouring sites
*wR*(*F*^2^) = 0.164	H-atom parameters constrained
*S* = 1.07	*w* = 1/[σ^2^(*F*_o_^2^) + (0.1305*P*)^2^ + 0.5925*P*] where *P* = (*F*_o_^2^ + 2*F*_c_^2^)/3
2432 reflections	(Δ/σ)_max_ = 0.001
163 parameters	Δρ_max_ = 1.78 e Å^−^^3^
0 restraints	Δρ_min_ = −1.28 e Å^−^^3^

### Special details

**Table:**

Geometry. All e.s.d.'s (except the e.s.d. in the dihedral angle between two l.s. planes) are estimated using the full covariance matrix. The cell e.s.d.'s are taken into account individually in the estimation of e.s.d.'s in distances, angles and torsion angles; correlations between e.s.d.'s in cell parameters are only used when they are defined by crystal symmetry. An approximate (isotropic) treatment of cell e.s.d.'s is used for estimating e.s.d.'s involving l.s. planes.
Refinement. Refinement of *F*^2^ against ALL reflections. The weighted *R*-factor *wR* and goodness of fit *S* are based on *F*^2^, conventional *R*-factors *R* are based on *F*, with *F* set to zero for negative *F*^2^. The threshold expression of *F*^2^ > σ(*F*^2^) is used only for calculating *R*-factors(gt) *etc*. and is not relevant to the choice of reflections for refinement. *R*-factors based on *F*^2^ are statistically about twice as large as those based on *F*, and *R*- factors based on ALL data will be even larger.

### Fractional atomic coordinates and isotropic or equivalent isotropic displacement parameters (Å^2^)

**Table:**

	*x*	*y*	*z*	*U*_iso_*/*U*_eq_	
Br1A	−0.10431 (6)	0.72017 (5)	0.94001 (2)	0.0266 (2)	
Cl1A	0.58277 (16)	−0.04318 (14)	0.11075 (6)	0.0269 (3)	
O1A	0.7080 (5)	0.2207 (4)	0.47588 (19)	0.0270 (6)	
C12A	−0.0341 (6)	0.5947 (5)	0.7495 (3)	0.0205 (7)	
H12A	−0.1777	0.6408	0.7378	0.025*	
C1A	0.3919 (6)	0.1019 (5)	0.3683 (3)	0.0195 (7)	
C2A	0.5227 (6)	0.0801 (5)	0.2923 (3)	0.0214 (7)	
H2AA	0.6756	0.1315	0.2992	0.026*	
C11A	0.0849 (6)	0.5125 (5)	0.6764 (3)	0.0215 (7)	
H11A	0.0215	0.5019	0.6146	0.026*	
C5A	0.0776 (6)	−0.0794 (5)	0.2713 (3)	0.0232 (8)	
H5AA	−0.0727	−0.1364	0.2645	0.028*	
C10A	0.2967 (6)	0.4452 (5)	0.6929 (3)	0.0207 (7)	
C8A	0.3490 (7)	0.2931 (6)	0.5299 (3)	0.0245 (8)	
H8AA	0.1944	0.3044	0.5110	0.029*	
C14A	0.2701 (6)	0.5451 (5)	0.8595 (3)	0.0229 (7)	
H14A	0.3318	0.5556	0.9216	0.027*	
C3A	0.4245 (6)	−0.0180 (5)	0.2068 (3)	0.0200 (7)	
C15A	0.3894 (6)	0.4648 (5)	0.7854 (2)	0.0210 (7)	
H15A	0.5352	0.4227	0.7974	0.025*	
C13A	0.0594 (7)	0.6090 (5)	0.8404 (2)	0.0200 (7)	
C6A	0.1690 (6)	0.0229 (5)	0.3577 (3)	0.0214 (7)	
H6AA	0.0799	0.0386	0.4091	0.026*	
C7A	0.5018 (6)	0.2071 (5)	0.4605 (2)	0.0213 (7)	
C9A	0.4280 (6)	0.3546 (5)	0.6187 (3)	0.0210 (7)	
H9AA	0.5826	0.3381	0.6351	0.025*	
C4A	0.2037 (6)	−0.0988 (5)	0.1951 (3)	0.0228 (7)	
H4AA	0.1399	−0.1661	0.1360	0.027*	

### Atomic displacement parameters (Å^2^)

**Table:**

	*U*^11^	*U*^22^	*U*^33^	*U*^12^	*U*^13^	*U*^23^
Br1A	0.0240 (3)	0.0339 (3)	0.0202 (3)	0.00673 (19)	0.00381 (18)	0.0001 (2)
Cl1A	0.0292 (5)	0.0316 (5)	0.0200 (5)	0.0051 (4)	0.0061 (3)	0.0036 (4)
O1A	0.0217 (13)	0.0343 (15)	0.0224 (14)	0.0029 (12)	0.0001 (10)	0.0010 (11)
C12A	0.0183 (16)	0.0213 (17)	0.0211 (17)	−0.0020 (13)	−0.0003 (13)	0.0044 (14)
C1A	0.0205 (17)	0.0196 (17)	0.0191 (17)	0.0030 (13)	0.0014 (13)	0.0057 (13)
C2A	0.0207 (17)	0.0211 (17)	0.0213 (17)	0.0032 (14)	−0.0002 (13)	0.0026 (14)
C11A	0.0230 (17)	0.0209 (17)	0.0195 (17)	−0.0015 (14)	−0.0015 (13)	0.0039 (13)
C5A	0.0174 (16)	0.0211 (17)	0.030 (2)	−0.0020 (13)	−0.0039 (14)	0.0061 (15)
C10A	0.0222 (18)	0.0199 (17)	0.0194 (17)	−0.0032 (14)	−0.0003 (14)	0.0050 (13)
C8A	0.0228 (18)	0.0278 (19)	0.0217 (18)	0.0016 (14)	0.0005 (14)	0.0033 (15)
C14A	0.0227 (18)	0.0233 (18)	0.0213 (17)	−0.0006 (14)	−0.0019 (14)	0.0036 (14)
C3A	0.0201 (17)	0.0211 (18)	0.0197 (17)	0.0058 (13)	0.0029 (13)	0.0047 (14)
C15A	0.0179 (16)	0.0258 (18)	0.0192 (17)	0.0022 (13)	0.0018 (13)	0.0045 (14)
C13A	0.0252 (18)	0.0181 (17)	0.0155 (17)	0.0027 (14)	0.0045 (14)	−0.0006 (13)
C6A	0.0208 (17)	0.0239 (18)	0.0208 (18)	0.0013 (14)	0.0034 (13)	0.0075 (14)
C7A	0.0239 (17)	0.0218 (17)	0.0187 (17)	0.0006 (14)	0.0017 (14)	0.0061 (14)
C9A	0.0203 (17)	0.0208 (17)	0.0218 (18)	−0.0003 (14)	0.0008 (14)	0.0055 (14)
C4A	0.0240 (18)	0.0218 (17)	0.0203 (17)	0.0035 (14)	−0.0043 (14)	0.0008 (14)

### Geometric parameters (Å, °)

**Table:**

Br1A—C13A	1.896 (4)	C5A—H5AA	0.9500
Cl1A—C3A	1.747 (4)	C10A—C15A	1.413 (5)
O1A—C7A	1.219 (5)	C10A—C9A	1.463 (5)
C12A—C11A	1.388 (6)	C8A—C9A	1.339 (5)
C12A—C13A	1.398 (5)	C8A—C7A	1.487 (5)
C12A—H12A	0.9500	C8A—H8AA	0.9500
C1A—C6A	1.395 (5)	C14A—C13A	1.387 (5)
C1A—C2A	1.402 (5)	C14A—C15A	1.396 (5)
C1A—C7A	1.503 (5)	C14A—H14A	0.9500
C2A—C3A	1.387 (5)	C3A—C4A	1.387 (5)
C2A—H2AA	0.9500	C15A—H15A	0.9500
C11A—C10A	1.396 (5)	C6A—H6AA	0.9500
C11A—H11A	0.9500	C9A—H9AA	0.9500
C5A—C4A	1.388 (6)	C4A—H4AA	0.9500
C5A—C6A	1.395 (5)		
			
C11A—C12A—C13A	119.4 (3)	C15A—C14A—H14A	120.7
C11A—C12A—H12A	120.3	C2A—C3A—C4A	122.0 (3)
C13A—C12A—H12A	120.3	C2A—C3A—Cl1A	119.4 (3)
C6A—C1A—C2A	120.2 (3)	C4A—C3A—Cl1A	118.6 (3)
C6A—C1A—C7A	121.8 (3)	C14A—C15A—C10A	121.1 (3)
C2A—C1A—C7A	117.9 (3)	C14A—C15A—H15A	119.5
C3A—C2A—C1A	118.7 (3)	C10A—C15A—H15A	119.5
C3A—C2A—H2AA	120.7	C14A—C13A—C12A	121.5 (3)
C1A—C2A—H2AA	120.7	C14A—C13A—Br1A	119.2 (3)
C12A—C11A—C10A	120.8 (3)	C12A—C13A—Br1A	119.3 (3)
C12A—C11A—H11A	119.6	C1A—C6A—C5A	119.6 (3)
C10A—C11A—H11A	119.6	C1A—C6A—H6AA	120.2
C4A—C5A—C6A	120.7 (3)	C5A—C6A—H6AA	120.2
C4A—C5A—H5AA	119.6	O1A—C7A—C8A	122.6 (3)
C6A—C5A—H5AA	119.6	O1A—C7A—C1A	120.2 (3)
C11A—C10A—C15A	118.7 (4)	C8A—C7A—C1A	117.2 (3)
C11A—C10A—C9A	123.1 (3)	C8A—C9A—C10A	125.6 (4)
C15A—C10A—C9A	118.2 (3)	C8A—C9A—H9AA	117.2
C9A—C8A—C7A	120.4 (4)	C10A—C9A—H9AA	117.2
C9A—C8A—H8AA	119.8	C3A—C4A—C5A	118.8 (3)
C7A—C8A—H8AA	119.8	C3A—C4A—H4AA	120.6
C13A—C14A—C15A	118.5 (3)	C5A—C4A—H4AA	120.6
C13A—C14A—H14A	120.7		
			
C6A—C1A—C2A—C3A	1.2 (5)	C7A—C1A—C6A—C5A	−177.6 (3)
C7A—C1A—C2A—C3A	179.4 (3)	C4A—C5A—C6A—C1A	−1.9 (6)
C13A—C12A—C11A—C10A	−0.4 (6)	C9A—C8A—C7A—O1A	−14.5 (6)
C12A—C11A—C10A—C15A	−0.9 (6)	C9A—C8A—C7A—C1A	166.1 (4)
C12A—C11A—C10A—C9A	179.0 (3)	C6A—C1A—C7A—O1A	155.6 (4)
C1A—C2A—C3A—C4A	−1.7 (5)	C2A—C1A—C7A—O1A	−22.6 (5)
C1A—C2A—C3A—Cl1A	178.8 (3)	C6A—C1A—C7A—C8A	−25.0 (5)
C13A—C14A—C15A—C10A	−1.3 (6)	C2A—C1A—C7A—C8A	156.8 (3)
C11A—C10A—C15A—C14A	1.7 (6)	C7A—C8A—C9A—C10A	178.5 (3)
C9A—C10A—C15A—C14A	−178.1 (3)	C11A—C10A—C9A—C8A	−13.4 (6)
C15A—C14A—C13A—C12A	0.0 (6)	C15A—C10A—C9A—C8A	166.4 (4)
C15A—C14A—C13A—Br1A	−179.5 (3)	C2A—C3A—C4A—C5A	0.4 (5)
C11A—C12A—C13A—C14A	0.8 (6)	Cl1A—C3A—C4A—C5A	179.8 (3)
C11A—C12A—C13A—Br1A	−179.7 (3)	C6A—C5A—C4A—C3A	1.4 (6)
C2A—C1A—C6A—C5A	0.6 (5)		

### Hydrogen-bond geometry (Å, °)

**Table:**

Cg1 is the centroid of the C1A–C6A ring and Cg2 is the centroid of the C10A–C15A ring.

**Table:**

*D*—H···*A*	*D*—H	H···*A*	*D*···*A*	*D*—H···*A*
C2A—H2AA···Cg2^i^	0.95	2.97	3.588 (4)	124
C5A—H5AA···Cg2^ii^	0.95	2.84	3.463 (4)	124
C12A—H12A···Cg1^iii^	0.95	2.83	3.527 (4)	131

Symmetry codes: (i) −*x*+1, −*y*+1, −*z*+1; (ii) −*x*, −*y*, −*z*+1; (iii) −*x*, −*y*+1, −*z*+1.
